# The Combination of Carmustine Wafers and Fotemustine in Recurrent Glioblastoma Patients: A Monoinstitutional Experience

**DOI:** 10.1155/2014/678191

**Published:** 2014-04-09

**Authors:** Giuseppe Lombardi, Alessandro Della Puppa, Fable Zustovich, Ardi Pambuku, Patrizia Farina, Pasquale Fiduccia, Anna Roma, Vittorina Zagonel

**Affiliations:** ^1^Medical Oncology 1 Unit, Venetian Oncology Institute-IRCCS, Via Gattamelata 64, 35128 Padua, Italy; ^2^Neurosurgery Department, Azienda Ospedaliera di Padova, 35128 Padua, Italy; ^3^Clinical Trials and Biostatistics Unit, Venetian Oncology Institute-IRCCS, 35128 Padua, Italy

## Abstract

*Background*. To date, there is no standard treatment for recurrent glioblastoma. We analyzed the feasibility of second surgery plus carmustine wafers followed by intravenous fotemustine. * Methods*. Retrospectively, we analyzed patients with recurrent glioblastoma treated with this multimodal strategy. * Results*. Twenty-four patients were analyzed. The median age was 53.6; all patients had KPS between 90 and 100; 19 patients (79%) performed a gross total resection > 98% and 5 (21%) a gross total resection > 90%. The median progression-free survival from second surgery was 6 months (95% CI 3.9–8.05) and the median OS was 14 months (95% CI 11.1–16.8 months). Toxicity was predominantly haematological: 5 patients (21%) experienced grade 3-4 thrombocytopenia and 3 patients (12%) grade 3-4 leukopenia. * Conclusion*. This multimodal strategy may be feasible in patients with recurrent glioblastoma, in particular, for patients in good clinical conditions.

## 1. Introduction

Malignant gliomas account for approximately 50% of all malignant primary brain tumors in adults and glioblastoma is the most common glial tumor. And so glioblastomas are relatively rare tumors and although the prognosis has improved in the last years, the survival is still poor. Standard therapy for newly diagnosed glioblastoma includes surgical resection when feasible, radiotherapy, and temozolomide according to Stupp regimen [1]. However, despite optimal treatment, median survival ranges from 12 to 15 months for glioblastoma [1]. Regarding second-line treatment in patients with recurrent glioblastoma there is no standard therapy. In the last years, there was interest in the role of bevacizumab, alone or in combination with cytotoxic drugs, but the results were conflicting [2–5]. Moreover, various antineoplastic agents, such as procarbazine, carmustine, lomustine, vincristine, rechallenge with temozolomide, and some combinations of those, were used. Numerous retrospective and prospective phase II studies showed an important activity of fotemustine in recurrent glioblastoma [6, 7] with a range of median overall survival from start of fotemustine treatment from 6 months to 11 months [7, 8]. Fotemustine (diethyl 1-{1-[3-(2-chloroethyl)-3-nitrosoureido] ethyl} phosphonate) is an alkylating [9] cytotoxic agent, belonging to the group of nitrosourea. It is characterized by elevated lipophilic properties and a low molecular weight that contribute to facilitation of its passage through the blood-brain barrier [10]. Moreover, fotemustine shows an important diffusion in neuronal cells and glia. The antitumor activity of fotemustine is related to its ability to alkylate DNA. After intravenous infusion, the plasma concentration reached the steady state in 45 minutes and the plasma concentration varied between 1 and 14 ug/mL disappearing in the blood within three hours [9].

In recent years, advances in surgery and the introduction of local chemotherapy have also provided modest improvements in survival. Local chemotherapy with 1,3-bis(2-chloroethyl)-1-nitrosourea (BCNU or carmustine) wafer has the advantage of bypassing the blood-brain barrier, delivering carmustine directly to peritumoral tissue and avoiding systemic toxicity; noteworthy, studies with carmustine wafers showed an overall survival benefit in newly diagnosed and recurrent malignant glioma [11, 12].

However, it is necessary to consider multimodal strategies that maximize the potency of available treatments through synergistic effects, for example, the use of local chemotherapy with carmustine and systemic treatment.

And so, the objective of this retrospective study is to analyse the feasibility of combination treatment with carmustine wafers and fotemustine in patients with recurrent glioblastoma treated at our oncological center.

## 2. Methods

In this retrospective study, we analyzed all patients with recurrent glioblastoma treated at our Oncological Center, Venetian Oncology Institute, and Department of Neurosurgery, Azienda Ospedaliera di Padova, with carmustine wafer positioned during second surgery and subsequently systemic treatment with fotemustine, from October 2008 to July 2013. Patients were eligible for this study if they had recurrent glioblastoma after standard first line therapy with temozolomide and radiotherapy, carmustine wafer positioned during second surgery, and fotemustine drug as second-line treatment performed between 14 and 21 days after the second surgery. Intravenous fotemustine was administrated at 100 mg/m^2^ every week for 3 consecutive weeks followed by a 5-week rest period; subsequently, an infusion was done every 3 weeks until progression of the disease or unacceptable toxicity or until a maximum of 12 cycles was reached. Fotemustine toxicity was graduated by National Cancer Institute-Common Toxicity Criteria scale version 4.0 (CTCAE v4.0). Surgical outcome was evaluated by assessing the resection rate as gross total resection (GTR) (GTR > 98% and GTR > 90%). Tumor location and extent of resection were defined by an expert neuroradiologist based on pre- and postoperative magnetic resonance imaging (MRI). During fotemustine therapy tumor response was evaluated by clinician assessment and by MRI according to Macdonald criteria [13] every two months or when clinically indicated. Physical examinations, full blood counts, and blood chemistry including hepatic and renal function tests were performed before each fotemustine infusion.

We analyzed clinical outcome in terms of median progression-free survival (PFS) and median overall survival (OS). Progression-free survival was calculated from second surgery until disease progression or death from any cause or to the last day of followup if alive. Overall survival (OS) was calculated from second surgery to the date of death from any cause or to the last day of followup if alive. PFS and OS were described using Kaplan-Meier survival curves and the log-rank test was used for group comparisons. *P* values were based on 2-side testing, and differences with a *P* ≤ 0.05 were considered significant.

All statistical analyses were performed using SPSS version 15.0 statistical software (SPSS Inc., Chicago, IL, USA).

## 3. Results

During the inclusion period, 24 consecutive patients with recurrent glioblastoma treated with carmustine wafers and intravenous fotemustine were recognised (see Table 1): thirteen patients were males (54%) and nine females (46%); median age was 54 (range 32–77). Karnofsky performance status (KPS) was 90–100 in all patients. Nineteen patients (79%) had a GTR > 98% and 5 patients (21%) had a GTR > 90%. Seventeen patients were available for* MGMT* gene analysis and 10 (59%) patients had methylated* MGMT* gene promoter. At the time of analysis, 2 patients were still alive.

**Figure 1:**
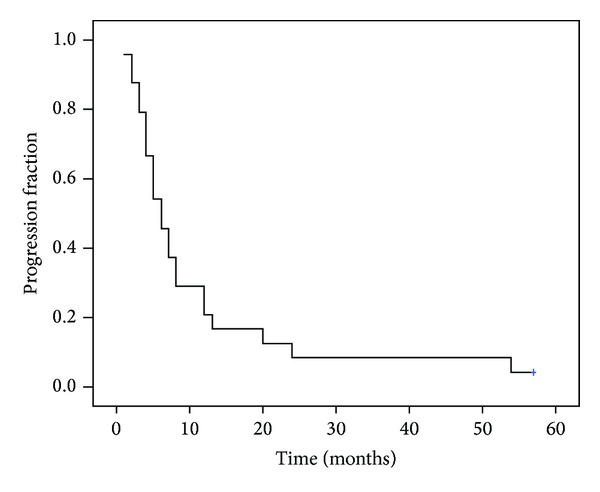
Kaplan-Meier progression free survival (PFS) curve; median PFS from second surgery: 6 months (95% CI 3.9–8.05).

**Figure 2:**
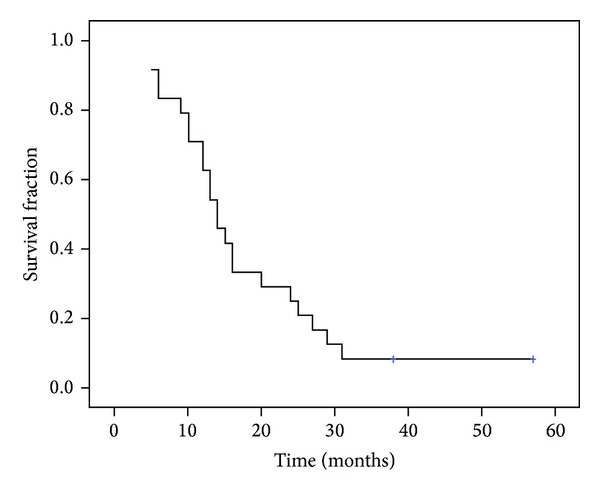
Kaplan-Meier overall survival (OS) curve; median OS from second surgery: 14 months (95% CI 11.1–16.8 months).

The median time from first and second surgery was 17 months. Among all patients, the median PFS from second surgery was 6 months (95% CI 3.9–8.05) and the median OS was 14 months (95% CI 11.1–16.8 months), as shown in Figures 1 and 2. Median survival from the first diagnosis of disease was 34 months (95% CI 24.3–43.9). No significant association was found between the resection rate (GTR > 98% versus GTR > 90%) and PFS (*P* = 0.4) and OS (*P* = 0.9).

All patients were evaluable for toxicity (see Table 2) which was predominantly haematological; in fact, grade 3-4 haematological toxicity was experienced by 8 patients (33%): 5 patients (21%) with grade 3-4 thrombocytopenia and 3 patients (12%) with grade 3-4 leukopenia. Among nonhaematological toxicity, 5 patients (21%) had grade 1-2 asthenia, 2 patients (8%) had grade 1-2 nausea/vomiting, and 2 patients (8%) reported hypertransaminasemia.

## 4. Discussion

Glioblastoma, the most common primary malignant brain tumor in adults, is associated with a poor prognosis. In particular, this tumor presents high local recurrence rate after the first treatment and patients with relapse have a very short survival. Presently, there is no consensus therapy recommended for the treatment of recurrent glioblastoma; a second debulking surgery could be proposed as well as a second line of chemotherapy. Various antineoplastic agents, such as procarbazine, carmustine, lomustine, vincristine, or some combinations of those, were used. Moreover, various phase II studies showed an important activity of fotemustine in recurrent glioblastoma [6]. Three studies used the standard schedule of fotemustine: three weekly doses (100 mg/m^2^) of fotemustine (induction phase) followed, after a 5-week rest, by fotemustine (100 mg/m^2^) every 3 weeks (maintenance phase) [14–16] showing a median PFS of 1.7–6.1 months and a median OS of 6–9.1 months. The most important toxicity was haematological: 8–15% of patients developed grade 3-4 thrombocytopenia and 4–10% grade 3-4 leukopenia. Addeo et al. [7], in a prospective single-arm phase II study, tested a new schedule of fotemustine in temozolomide-pretreated patients with glioblastoma; all patients underwent fotemustine 80 mg/m^2^ every 2 weeks for five consecutive administrations (induction phase) and then every 4 weeks at 80 mg/m^2^ as maintenance. This schedule was generally well tolerated with a good activity; in fact, the median PFS and OS were 6.7 and 11 months, respectively. Moreover, only 7% and 3% of patients developed grade 3 thrombocytopenia and leukopenia, respectively.

On the other side, the extent of resection for recurrent glioblastoma has been shown to have an impact on overall survival; recently, Bloch et al. [17] analyzed 107 patients with resections for recurrent glioblastoma; they demonstrated that the extent of resection at recurrence is an important predictor of overall survival and if gross total resection is achieved at recurrence, overall survival is maximized regardless of initial extent of resection, suggesting that patients with initial subtotal resection may benefit from surgery with a gross total resection at recurrence.

Recently, Quick et al. [18] performed a retrospective study analyzing the benefit of tumor resection for recurrent glioblastoma. They analyzed 40 patients and a radiologically confirmed complete resection was achieved in 29 patients (72.5%); median followup was 18.8 months, and median survival after re-resection was 13.5 months. They demonstrated that only complete removal of contrast enhancing tumor was significantly correlated with survival after re-resection according to multivariate analysis; in contrast, time between first diagnosis and tumor-recurrence, tumor volume at recurrence, and MGMT status were not significantly correlated with survival after second surgery. They concluded that recurrent glioblastoma patients in good clinical condition should be treated with second surgery. In our study, we did not analyze the predictive role of MGMT due to having few patients.

Noteworthy, local chemotherapy with carmustine wafer has demonstrated an overall survival benefit both in newly diagnosed and recurrent glioblastoma [11, 12]; in particular, in a placebo-controlled, double-blind, randomized, prospective phase 3 trial Westphal et al. [11], using a Cox proportional hazards model, demonstrated that carmustine wafers significantly prolonged survival in newly diagnosed glioblastoma patients (*P* = 0.04), with a risk reduction of 31% (95% CI 3%–51%). Moreover, Brem et al. [11, 12] showed that carmustine wafer has a significant beneficial effect (hazard ratio 0.67, *P* = 0.02) in recurrent glioblastoma, as well.

To our knowledge, this is the first study analyzing the multimodal strategy with resection plus carmustine wafers plus systemic therapy with fotemustine in patients with recurrent glioblastoma. We demonstrated an interesting benefit of this multimodal treatment with a median PFS of 6 months and a median OS of 14 months. Notably, all the patients were in very good clinical conditions, all patients performed a gross total resection, and the majority of patients had MGMT methylated. Toxicity was slightly higher than expected, and the greater frequency of adverse events was during the induction phase. Carmustine is released in a controlled manner over approximately 3–6 weeks and so, likely, it may have increased the fotemustine toxicity. However, the most important toxicity was haematological and it was always manageable, as well. Moreover, employing a fotemustine schedule with a lower toxicity, for example, the fotemustine schedule used by Addeo et al. [7], the treatment may be more feasible.

In conclusion, the multimodal strategy with gross total resection plus carmustine wafers plus intravenous fotemustine administration may be feasible in patients with recurrent glioblastoma; in particular, this treatment should be considered for patients in good clinical conditions.

## Figures and Tables

**Table 1:** Characteristics of the patients.

Characteristics	*N*. (%)
Patients	24
Sex	
Male	13 (54%)
Female	9 (46%)
Median age (years)	54 (32–77)
KPS	90–100
Histology	
Glioblastoma	100%
PTS with GTR >98%	19 (79%)
PTS with GTR >90%	5 (21%)
PTS with methylated MGMT	10/17 (59%)
PTS with unmethylated MGMT	7/17 (41%)

KPS: Karnofsky performance status; MGMT: O6-methylguanine-DNA methyltransferase; PTS: patients; GTR: gross-total resection.

**Table 2:** Overall toxicity during all treatment courses by type and grade.

Toxicity	Grades 1-2	Grades 3-4
Haematological toxicity		
Thrombocytopenia	11 (46%)	5 (21%)
Neutropenia	4 (17%)	3 (12%)
Anemia	1 (4%)	0
Nonhaematological toxicity		
Nausea/vomiting	2 (8%)	0
hypertransaminasemia	5 (21%)	0
Asthenia	2 (8%)	0
